# Supplementary material for: Isolation and Purification-Free Digital Single-Small Extracellular Vesicle Biosensing with Scalable Plasmonic Arrays
Source: bioRxiv. 2026 May 4:2026.04.30.721846. Preprint. [Version 1] doi: 10.64898/2026.04.30.721846 (PMC13174645; doi:10.64898/2026.04.30.721846)
Supplement: Supplement 1 [file media-1.docx]

Isolation and Purification-Free Digital Single-Small Extracellular Vesicle Biosensing with Scalable Plasmonic Arrays

*Mohammad Sadman Mallick^1,†^, Saswat Mohapatra^1,†^, Abhay Kotnala^1^, A B M Arafat Hossain^1^, and Wei-Chuan Shih^1,2,3,4,*^*

^1^Department of Electrical and Computer Engineering, University of Houston, 4800 MLK Blvd., Houston, Texas 77204, United States of America

^2^Department of Biomedical Engineering, University of Houston, 4800 MLK Blvd., Houston, Texas 77204, United States of America

^3^Department of Chemistry, University of Houston, 4800 MLK Blvd., Houston, Texas 77204, United States of America

^4^Program of Materials Science and Engineering, University of Houston, 4800 MLK Blvd., Houston, Texas 77204, United States of America

**^*^E-mail:** wshih@uh.edu

^†^These authors contributed equally.

**Supplementary Note 1:**

**Preparation of Polystyrene Beads and Substrates**

Polystyrene beads (PSB) were first cleaned to remove surfactants and increase hydrophilicity. The beads were rinsed in a water–ethanol (1:1) mixture, followed by 6–7 centrifugation cycles and drying. The cleaned beads were then dispersed in a water–ethanol (1:2) solution by adding 100 µL of solvent per 1 mg of PSB.

Glass coverslips were cleaned sequentially in acetone and isopropanol baths, rinsed with copious DI water, and dried with nitrogen. A 2 nm Cr adhesion layer and 80 nm Au film were deposited by sputtering.

**Petri Dish Monolayer Assembly**

Approximately 250 µL of 460 nm PSB solution was dispensed onto the water surface using a syringe pump. This formed a white, turbid film at the air-water interface, indicating that the water surface was fully covered with a PSB monolayer. A drop of surfactant (sodium dodecyl sulfate) solution was added to a corner of the petri dish, which helped bring the PSB beads closer together. The substrate was then manually lifted horizontally using tweezers. Finally, the transferred film was dried on a hot plate at 70°C.

**Langmuir–Blodgett Trough (LBT) Method**

A commercial Langmuir-Blodgett trough (KSV NIMA) was employed. A monolayer film was formed by gently dispersing the PSB solution at the air-water interface using a syringe pump. Initially, the PSB particles moved randomly at the interface, gradually forming smaller monolayer domains. As more PSB solutions were added, the particles came closer together, creating a white, turbid film that indicated the water surface was fully covered with a PSB monolayer. The film was then compressed using robotic barriers, and surface pressure was monitored so that it doesn’t exceed 30 mN/m, promoting the formation of a continuous, hexagonally packed monolayer structure. The substrate, which had been previously immersed in the water inside the Langmuir-Blodgett trough, was then vertically pulled out, transferring the monolayer onto its surface while maintaining a constant surface pressure. Finally, the substrate was dried on a hot plate at 70°C to complete the process. The surface pressure was automatically regulated throughout the coating process using KSV NIMA software-controlled barriers to ensure uniformity.

**Nanosphere Settling Lithography (NSSL)**

A substrate was fully submerged in deionized (DI) water, followed by a similar setup to the petri dish method. Approximately 250 µL of PSB solution was then carefully dispensed onto the water surface using a syringe pump, creating a white, turbid film at the air-water interface. This film indicated the formation of a PSB monolayer. To improve the packing density of the polystyrene beads, a drop of sodium dodecyl sulfate (SDS) surfactant was added to one corner of the petri dish. This addition facilitated better organization of the beads by reducing surface tension. Gradual water removal via a drain at the petri dish bottom was initiated after the PSB monolayer had formed on the water surface. To regulate the drainage process, the hole was initially sealed with a transparent adhesive tape and then precisely punctured using a sharp pin, allowing controlled water removal. As the water level slowly lowered, the floating PSB monolayer gently settled onto the submerged substrate. The slow removal of water ensured a uniform deposition of the monolayer, minimizing defects during the transfer. Once the water was completely drained, leaving the PSB monolayer fully transferred onto the substrate, the coverslips were dried on a hot plate at 70°C to complete the NSSL process.

**PSB monolayer to AGNIS fabrication**

After the deposition of the 460 nm PSB monolayer onto the gold-coated glass coverslip substrates, oxygen plasma etching was employed to reduce the diameter to 360 nm. The etching parameters were set as follows: oxygen flow rate of 50 sccm, chamber temperature of 21°C, chamber pressure of 30 mTorr, and RF power of 50W. Argon milling was then performed to selectively remove the underlying gold unmasked by the PSBs, resulting in an arrayed gold nanodisk (AGN) under PSBs. The argon milling parameters included a normal angle of incidence, a sample holder distance of 30 cm, and an etch rate of 0.62 nm/s. The PSBs were then removed by clamping the glass coverslip substrate with a silicon wafer and the assembly was subjected to a 20-minute sonication process in water. After the removal of the PSBs, the gold nanodisks covered substrate was immersed in a buffered hydrofluoric acid solution for 85 sec to undercut the glass substrate beneath the disks, resulting in the final nanostructured plasmonic sensor, arrayed gold nanodisks on invisible substrates (AGNIS).

**Supplementary Note 2:**

The AGNIS chips fabricated were optically characterized by LSPR hyperspectral mapping and PANORAMA were performed using the optical setups described in Supplementary Figure S2a and 2b. Briefly, LSPR mapping was carried out on an inverted microscope coupled to a spectrometer for spatially resolved extinction spectra, and PANORAMA used a filtered halogen illumination path with a high-NA objective and sCMOS camera.

**
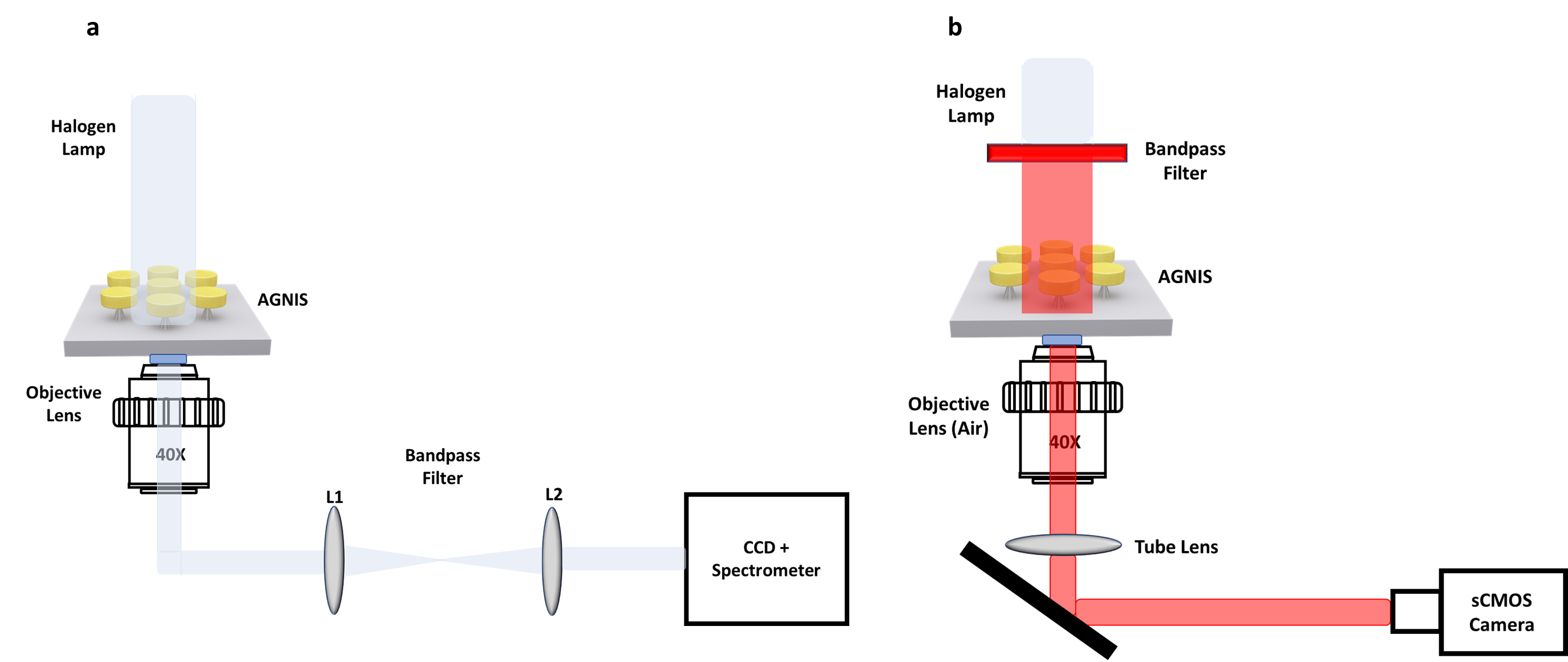
**

**Supplementary Figure S2:** Optical setup of **a.** LSPR mapping system **b.** PANORAMA.

**Supplementary Note 3:**

SEM imaging, performed at an accelerating voltage of 15 kV after sample drying and Au coating for conductivity, confirmed the binding of 100 nm PSBs to AGNIS (Supplementary Figure S3).


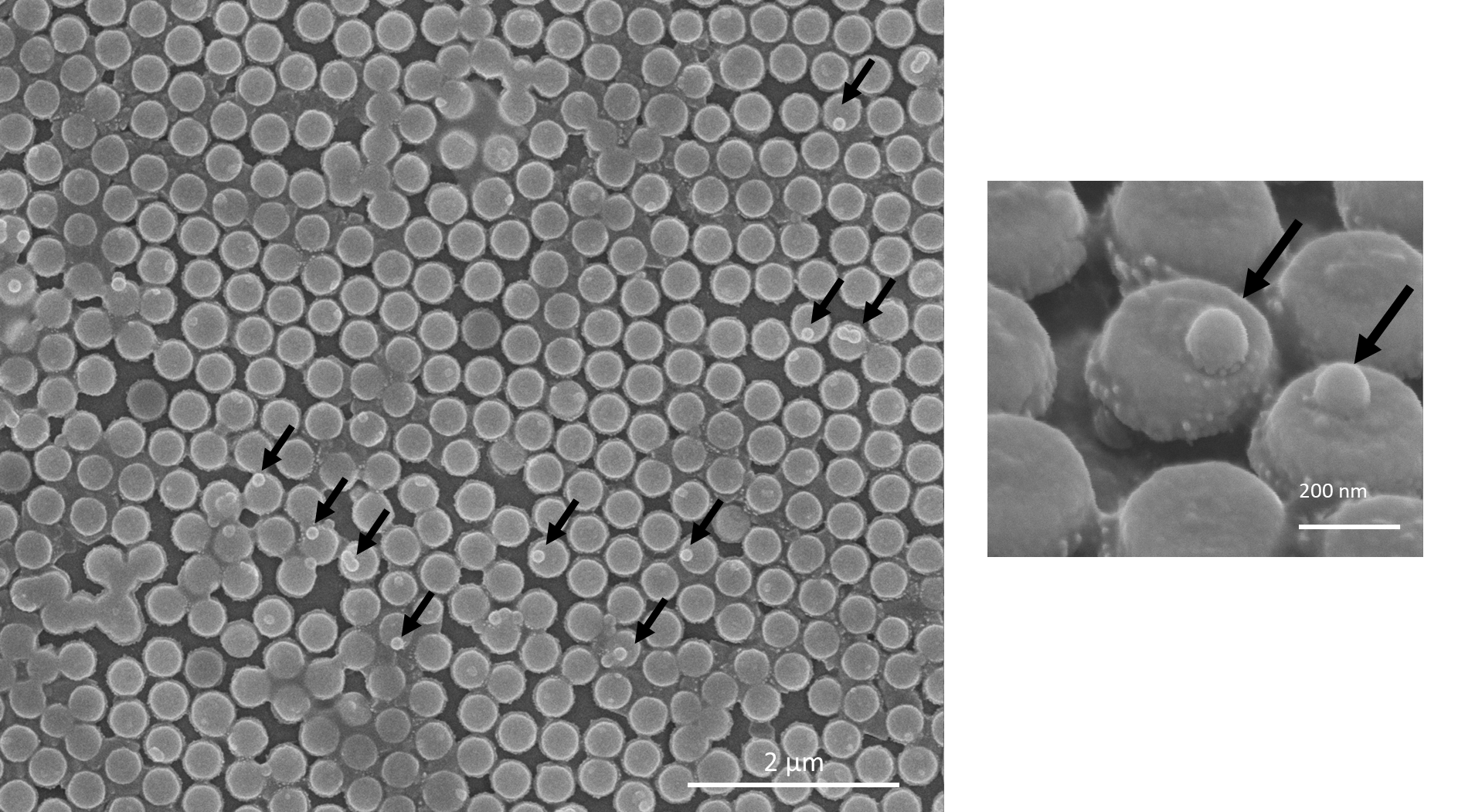


**Supplementary Figure S3.** SEM images of 100 nm PSBs settled on AGNIS.

**Supplementary Note 4:**

The washing protocol was optimized to ensure selective retention of bound sEVs while removing unbound or weakly attached vesicles. Specifically, the AGNIS chips were washed with 1× PBS for 0, 3, 5, 10, and 15 cycles. We observed that unbound sEVs were effectively removed by five wash cycles, beyond which the detected sEV count remained constant (Supplementary Figures S4a–b), indicating stable and specific capture. Supplementary Figure S4c–d shows representative PANORAMA images of detected particles and sEVs before and after washing at the 60 min time point on petri-dish and LBT substrates. SEM imaging was performed following vesicle fixation using a standard protocol^1^. SEM analysis confirmed a one-to-one sEV-to-nanodisk capture configuration, validating specific vesicle binding to individual AGNIS features (Supplementary Figure S4e).


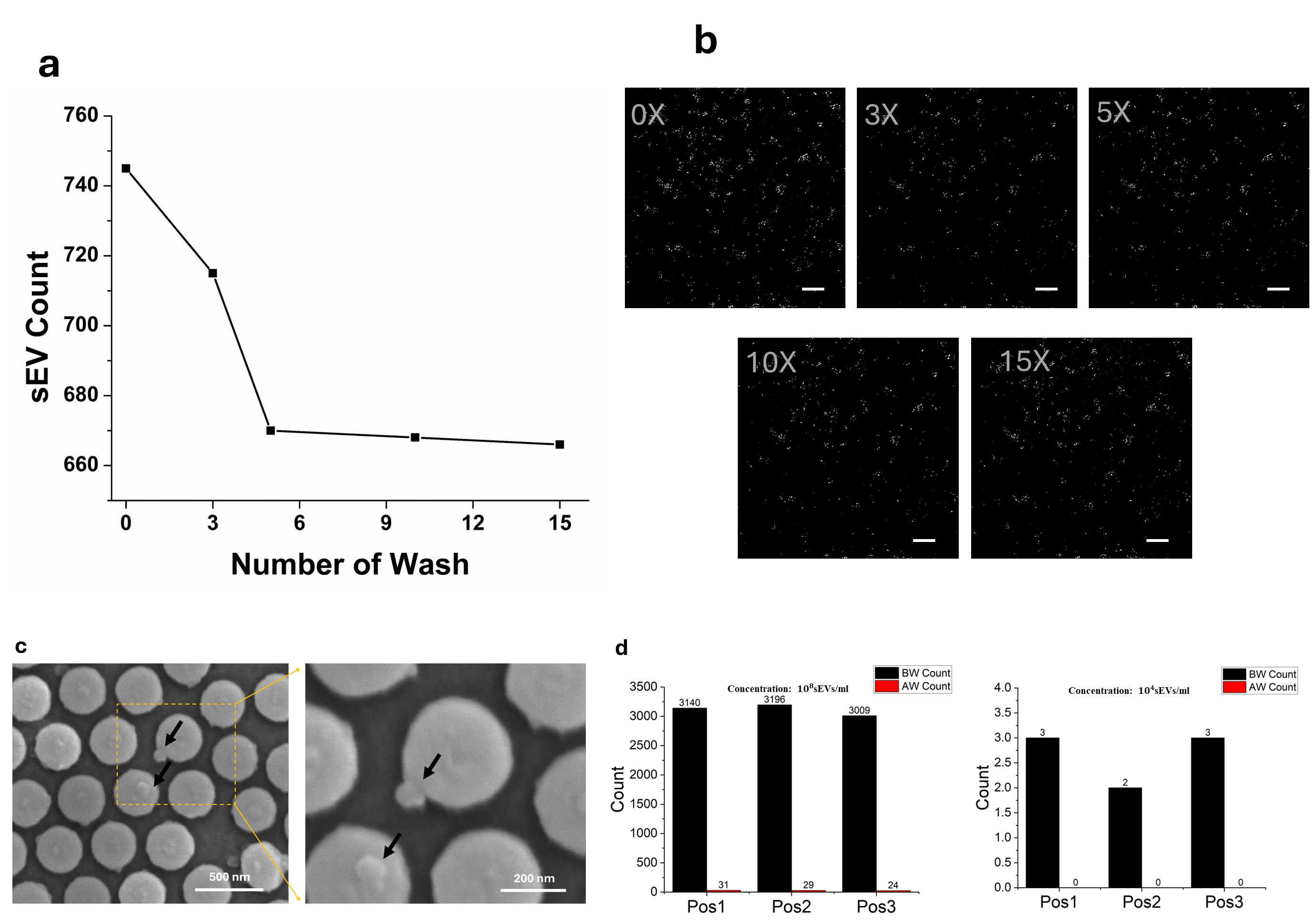


**Supplementary Figure S4. a-b.** Optimization of the washing step PANORAMA of detected sEVs isolated from H460 cancer cell line cultures using **c.** SEM images of sEVs on AGNIS after washing. **d.** Detected sEV counts at highest and lowest concentrations using biotinylated IgG in place of anti-CD9/CD63/CD81 antibodies for surface capture.

**References**

(1) Kapoor, K. S.; Kong, S.; Sugimoto, H.; Guo, W.; Boominathan, V.; Chen, Y.-L.; Biswal, S. L.; Terlier, T.; McAndrews, K. M.; Kalluri, R. Single Extracellular Vesicle Imaging and Computational Analysis Identifies Inherent Architectural Heterogeneity. *ACS Nano* **2024**, *18* (18), 11717–11731.
